# Violent Chorea St. Viti Cured by Strychnine

**Published:** 1845-12

**Authors:** 


*Violent Chorea St. Viti cured by Strychnine.*—Eliza Holstappen, aged 19, born in Germany, single. Entered the New York Hospital July 24, 1845. Is of large frame and robust appearance. Has had amenorrhœa four months, but otherwise has enjoyed good health, until about three weeks since her friends noticed a twitching of muscles. This increased until there was involuntary motion of all her limbs. Upon admission, she was unable to remain in bed, so that she was obliged to be kept upon the floor. Her bowels being opened, she was put upon Fowler's Solution, gtt. iv. ter in die. This was increased to every two hours by the fourth day, but her motions became more frequent and strong, so that she could not be restrained on the matress, and tore her clothes from her body. Her nights were sleepless, and she constantly screamed, although perfectly sensible.

On the 2d of August, she was put upon Carb. Ferri, which was continued for three days, the patient being at the same time freely purged with Croton and Castor Oil. This did not produce much benefit. As soon as evening came on, her motions became more and more convulsive, and her screams loud and incessant. For several nights in succession, she was obliged to be tied hand and foot to the bedstead, perfectly naked, as no covering could be kept on her. During the day she was more pacific.

On the 12th, we began the use of Pil. Strychnine, gr. 1-16, ter in die. The effect of this was almost immediate and very marked. It was continued four days, in the above quantity with evident improvement, her nights being more quiet, and some sleep obtained.

On the 16th, the pill was increased to 1 1-2 gr. This night she slept for an hour or more together, in a chair.

17th. Last night she slept in bed quietly for several hours, and this morning *was able to sew.* She walks about, although her motions are still violent. Has been on the use of the medicine just one week.

19th. The last two nights the patient has slept perfectly well during the whole night, without any noise; walks now tolerably straight; and visits the other wards. Her appetite is very great. During the whole of the attack, her mind has been entirely free from any delusion. She still continues the Strychnine ½ gr. ter in die, with progressive improvement. During the last two days she has occasionally complained of headache.

Sept. 1st. Our patient rapidly improved under this treatment, continued until within a few days, when she being apparently *well*, it was stopped, and no symptom of a relapse appearing, she was to-day *discharged cured.*

The pathology of Chorea, is among the mysteries of the science. The arsenical and ferruginous preparations, and drastic purgatives, which have either one or the other, generally succeeded in relieving the symptoms, having in this case entirely failed, the determination to try the Strychnine was made on the supposition of the condition of the nerves in this disease being analogous to that in Paralysis. In the latter case, there is a total loss of power over the muscles, in the other a partial loss only. If the rapid and felicitous result of the use of Strychnine in this case should lead to its further administration in Chorea, some light may perhaps be thrown on the pathology of the disease.—*Med. and Surg. Reporter.*

